# Quercetin as a Potential Modulator of P-Glycoprotein Expression and Function in Cells of Human Pancreatic Carcinoma Line Resistant to Daunorubicin

**DOI:** 10.3390/molecules15020857

**Published:** 2010-02-09

**Authors:** Sylwia Borska, Miroslaw Sopel, Magdalena Chmielewska, Maciej Zabel, Piotr Dziegiel

**Affiliations:** 1 Department of Histology and Embryology, Medical University, T. Chalubinski Street 6a, 50-368 Wroclaw, Poland; 2 Department of Histology and Embryology, University of Medical Sciences in Poznan, Swiecickiego Street 6, 60-781 Poznan, Poland; 3 Lower Silesian Centre of Oncology, Hirszfeld Square 12, 53-413 Wroclaw, Poland

**Keywords:** quercetin, daunorubicin, P-glycoprotein, multidrug resistance

## Abstract

P-glycoprotein (P-gp) is one of the ABC transporters responsible for the resistance of several tumours to successful chemotherapy. Numerous agents are capable of interfering with the P-gp-mediated export of drugs but unfortunately most of them produce serious side effects. Some plant polyphenols, including the flavonol quercetin (Q), manifest anti-neoplastic activity mainly due to their influence on cell cycle control and apoptosis. Reports are also available which show that Q may intensify action of cytostatic drugs and suppress the multidrug resistance (MDR) phenomenon. The study aimed at determination if Q sensitizes cells resistant to daunorubicin (DB) through its effect on P-gp expression and action. The experiments were conducted on two cell lines of human pancreatic carcinoma, resistant to DB EPP85-181RDB and sensitive EPP85-181P as a comparison. Cells of both lines were exposed to selected concentrations of Q and DB, and then membranous expression of P-gp and its transport function were examined. The influence on expression of gene for P-gp (*ABCB1*) was also investigated. Results of the studies confirmed that Q affects expression and function of P-gp in a concentration-dependent manner. Moreover it decreased expression of *ABCB1*. Thus, Q may be considered as a potential modulator of P-gp.

## Introduction

The phenomenon of multidrug resistance (MDR) was described for the first time by Kessel *et al*. in 1968. At present, insensitivity of neoplastic cells to chemotherapeutic agents is thought to represent one of the main causes of failure in treatment of tumour diseases. The best recognised and the most frequent cause of the resistance involves an increased activity of ATP-binding cassette family transporters (ABC) and is called ‘classical’ MDR [1]. Among membrane transporters, the overexpression most frequently involves P-glycoprotein (P-gp), encoded by the *ABCB1* gene, and has been closely associated with unfavourable prognostic index in cases of several types of tumours [2,3,4].

The substrates for P-gp represent substances of a variable structure, chemical properties and mechanisms of action [5]. Drugs affected by the MDR phenotype include several cytostatic drugs e.g., anthracyclines [6]. Daunorubicin (DB) which belongs to that group manifests a multi-directional mode of action. Its best characterized activities include an effect on activity of topoisomerase II (topo II) and interactions with DNA through formation of intercalation complexes, covalent bonds and modification of nucleic acid bases. This leads to disturbances in processes such as replication, transcription, DNA repair and most frequently results in cell apoptosis. Its involvement in generation of reactive oxygen species and lipid peroxidation has been also well documented [6,7,8]. Similarly to cases of other anti-neoplastic drugs, clinical application of DB is restricted due to its cytotoxic effects on normal cells. Within a year following termination of treatment with anthracyclines, the patients used to demonstrate cardiomyopathy and chronic cardiac failure. A bone marrow suppression and thrombosis were also described. In frequent cases MDR is developed often correlated with increased P-gp expression [8,9].

**Figure 1:**
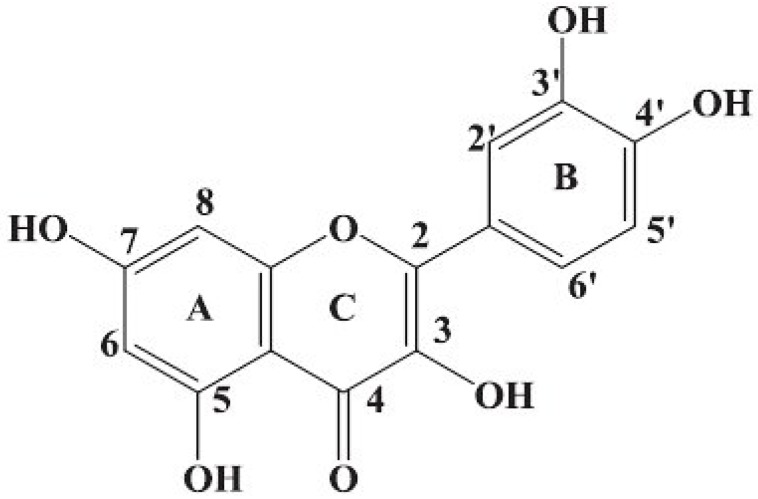
Structure of quercetin.

A large number of compounds have been identified that are capable of overruling MDR by interfering with the P-gp-mediated export of the drugs used for treatment. A search for P-gp modulators which would selectively inhibit activity of that transporter without any negative side effects has been continued [10,11]. Extensive hopes are linked to application of some plant polyphenols e.g., quercetin (Q). Flavonol Q (structure is shown in Figure 1) belongs to the large group of flavonoids, manifested in numerous edible plants. For several decades Q has been documented as a strong antioxidant, anti-inflammatory and vasodilating agent, decreasing blood pressure and inhibiting aggregation of blood platelets. Many data showed that Q might exert inhibitory effects on all stages of carcinogenesis [12,13,14,15]. Antineoplastic properties of Q are linked, first of all, to its effect on expression of some genes engaged in cell cycle control. Q exerts also a direct effect on activity of various type protein kinases significant in the process of carcinogenesis and the associated with them metabolic pathways [16,17,18,19,20]. Apart from its effect on induction of apoptosis in neoplastic cells, Q is considered to be effective in restoring drug sensitivity of MDR tumour cells. The results of *in vivo* and *in vitro* studies seem promising [21,22,23,24].

This study aimed at determination whether Q sensitizes cells of pancreatic carcinoma resistant cell line to action of DB by decreasing membraneous expression of P-gp, blocking its transport functions and affecting expression of *ABCB1 *gene.

## Results and Discussion

In our study two human pancreatic cancer cell lines, a resistant (RDB) and a sensitive (P) to DB, provided the cell models. We investigated the effects of Q against P-gp overexpression in cells with RDB fenotype and the comparison with P line as a control.

Cytotoxicity tests have demonstrated that Q inhibits cell divisions in both neoplastic cell lines in the concentration-related manner (Table 1, Table 2). Also the resistance factor (RF) for Q has approached 1 (RF = 1.25), which points to the lack of resistance to the compound. After 72 h of Q action IC_50_ for P cell line was estimated at 8 µM. In the case of RDB line IC_50 _amounted to 12 µM. For further experiments Q concentrations of 3, 6 and 12 µM were selected.

**Table 1:** Cytotoxic effect of Q on cells in DB-sensitive line as related to concentration of the drug after 72 h; p < 0.001 for optical density (OD) of C *vs*. all. C—Control.

Q concentration [µM]	C (0)	1	2	3	6	12	25	50
**OD**	2.472	2.363	2.175	1.930	1.596	0.650	0.351	0.273
**SD**	0.024	0.033	0.033	0.033	0.048	0.030	0.030	0.026
**% of C value **	100	95.584	87.999	78.074	64.556	26.281	14.186	11.057

**Table 2:** Cytotoxic effect of Q on cells of DB-resistant line, as related to drug concentration after 72h; p < 0.001 for optical density (OD) of C *vs*. all. C—Control.

Q [µM]	C (0)	1	2	3	6	12	25	50
**OD**	2.363	2.317	2.277	2.254	1.809	1.192	1.022	0.649
**SD**	0.035	0.119	0.021	0.059	0.013	0.008	0.058	0.039
**% of C value**	100	98.053	96.360	95.380	76.552	50.444	43.235	27.483

Many data show that Q manifests antiproliferative and pro-apoptotic properties, as documented in cases of multiple types of tumours. Additionally, results of studies suggest that Q may act synergistically with other anticancer substances and that it may sensitize some neoplastic cells to the apoptotic effect of cytostatic drugs [25,26,27]. Antiproliferative effect of Q on both pancreatic cell lines, augmentation of DB cytotoxic action in P cells and overcoming DB-resistance in RDB cells were proved in our studies.

**Figure 2:**
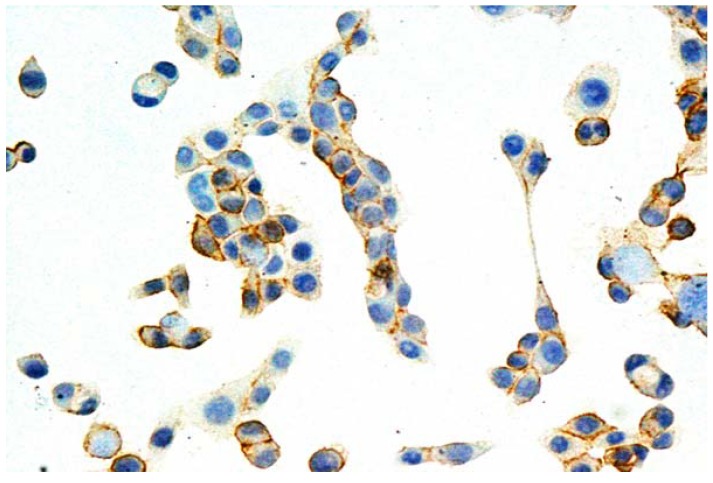
Expression of P-gp in membranes of cells in EPG85-257RDB line, subjected to action of Q12/K1. ×200.

The effect of Q on P-gp examined using immunocychemistry and Western blotting analysis proved that Q affects the protein expression level. Immunocytochemical staining demonstrated that in the P line of pancreatic carcinoma no P-gp could be detected in cell membranes, probably due to very low expression of that protein. In the case of RDB cell line the immunostaining showed presence of P-gp in cell membranes in almost 100% of cells. As compared to the control, a statistically significant decrease in the expression could be observed only after treatment with Q12, Q12/K1 (see Figure 2) and Q12/K2, and amounted to 20%, 22% and 23% respectively. However, the differences between Q12, Q12/K1 and Q12/K2 were insignificant (p > 0.05).

**Figure 3:**
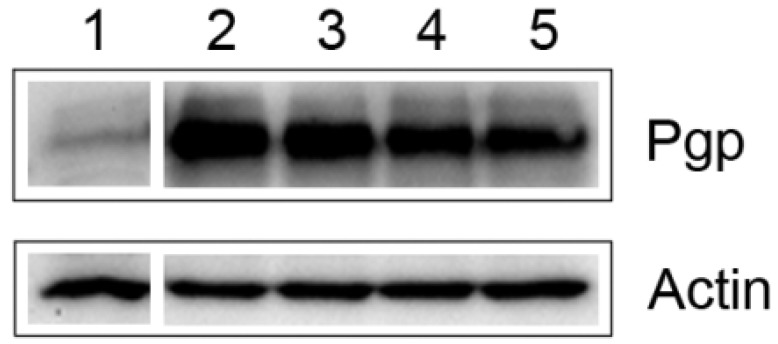
Western blotting analysis of P-gp expression after treatment with Q12 in RDB line. C219 detected 170kDa band in RDB line (lanes 2-5), revealing the decrease in P-gp expression level in RDB cell lines incubated with Q12 (lanes 4, 5). P cells (lane 1) were used as negative control for P-gp, and actin (42kDa) staining was used as loading control.

Western blot analyses confirmed tendency shown in the immunocytochemical stainings. After 72 h treatment of Q12 the amount of P-gp decreased in RDB cells of 30% (Figure 3).

**Figure 4:**
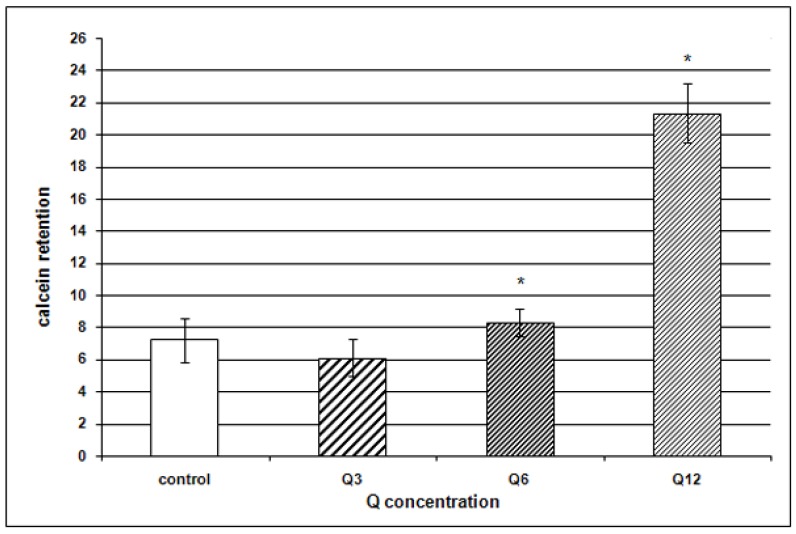
Retention of calcein in cells of EPP85-181RDB line following administration of various Q concentrations, as compared to the control; * p < 0.001 as compared to control.

Since P-gp expression level not always correlates with efficacy of drugs elimination from the cells, a functional test was also performed, evaluating P-gp transport function basing on measurement of calcein AM accumulation (CAM). The test with CAM is commonly applied in studies of this type and it is regarded to be effective [11,21,28]. CAM retention coefficient after administration of DB alone manifested no significant alterations in CAM retention, as compared to the control. We have demonstrated that Q12 decreased significantly P-gp transport functions in the RDB cells (Figure 4). A decrease of calcein retention (16%) was noted also following application of Q6, even if membranous expression of P-gp did not decrease significantly in this case. 

**Figure 5:**
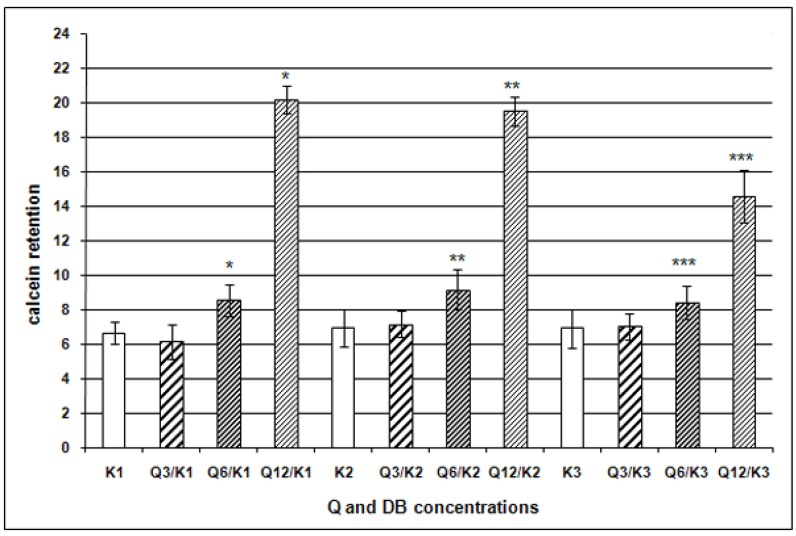
Retention of calcein in cells of EPP85-181RDB line following administration of Q and DB combination, as compared to action of DB alone; * p < 0.001 as compared to K1; ** p < 0.001 as compared to K2; *** p < 0.001 as compared to K3.

Using Q/DB combinations, increase in Q concentration at a stable concentration of DB was accompanied by a clear tendency for an increased calcein accumulation in RDB cells. The changes were statistically significant for combinations with Q6 and Q12 as compared to the action of DB alone (Figure 5). Taking into account increase in DB concentration at a stable concentration of Q we observed that it did not significantly affect calcein retention. Moreover, in combinations of Q12 with DB an increasing dose of DB was accompanied by a decreased fluorescence signal, in all probability due to a strong pro-apoptotic influence and a markedly reduced cell number (see Table 2; data about apoptosis not shown). Such phenomenon should be taken into account only if Q represented a substrate of P-gp and inhibition would take place by competition for the binding site. This was already suggested by some authors [28,29,30]. Nevertheless, our results related to cytotoxicity of Q to pancreatic carcinoma cells showed that Q cannot in this case represent a substrate of transporter proteins and the mechanism of its effects on P-gp transporter function is different. Several published reports indicate that Q represents a inhibitor of ATP-binding sites of P-gp [31,32,33]. It is also known that many inhibitors of ATP hydrolysis and factors affected *ABCB1* gen expression represent more effective modulators than P-gp substrates [10,26,32].

**Figure 6:**
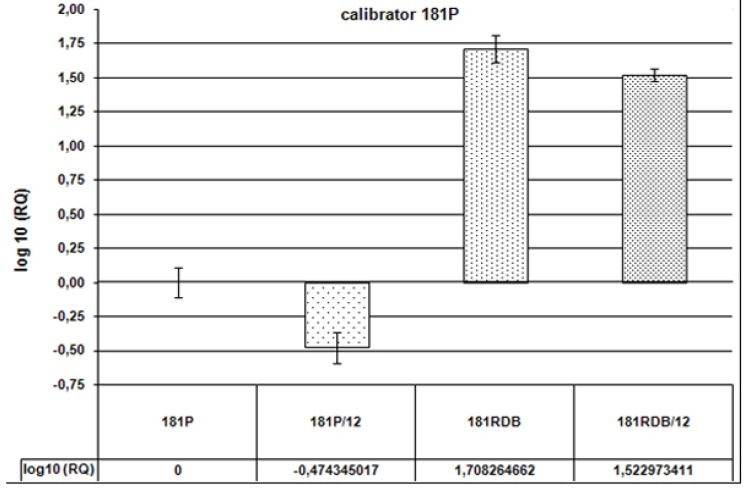
Expression of *ABCB1* gene—Relative analysis (RQ) as compared to the sensitive cell line (denoted as 181P). Values of RQ: for 181P = 1, for 181P/12 = 0.3355, for 181RDB = 51.08, for 181RDB/12 = 33.34. Calibrator: P line.

**Figure 7:**
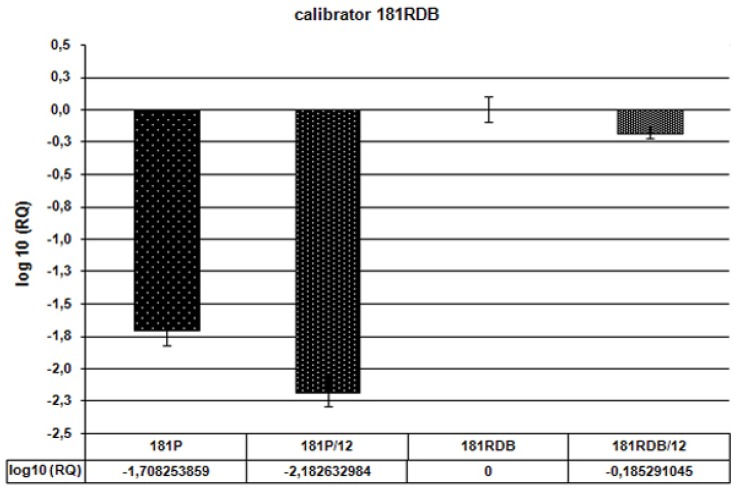
Expression of *ABCB1* gene—Relative analysis (RQ) as compared to the resistant cell line (denoted as 181RDB). Values of RQ: for 181P = 0.0195, for 181P/12 = 0.0065, for 181RDB = 1, for 181RDB/12 = 0.6526. Calibrator: RDB line.

In our studies the exposure to Q was sufficiently long to allow not only its direct effect on the protein but also control of its expression at the level of DNA/mRNA. Many reports indicated that polyphenols, including Q, may affect expression of P-gp encoding gene [11,22,34]. In our studies the real-time PCR has been employed to establish whether Q affects expression of *ABCB1* gene. Relative analysis of the results permitted to compare effects of Q12 on cells in P and RDB lines. We find out that expression of *ABCB1* gene in RDB cells is 51-fold higher than in the P line. After administration of Q12 it was only 33-fold higher (Figure 6). In RDB cells Q12 decreased the expression of *ABCB1* by 35%. Additionally, in the case of P cells Q12 decreased expression of the gene by 67% (Figure 7). 

The studies have focused on one of the most probable mechanisms of sensitizing the resistant cells to DB, through the effect of Q on P-gp expression and function. The flavonol was considered as a potential modulator of P-gp, affecting both the protein and the gene for it. Several reports indicated that Q might significantly help in overcoming MDR [1,2,21,22]. Some autors suggested that Q might also inhibit efflux of drugs from normal cells, e.g., in vascular endothelium, but only in high concentrations, most frequently exceeding 50 μM. On the other hand, lower doses may act reciprocally, intensifying elimination of toxic substances, which has a significant protective effect for normal tissues and organs [35]. Many data confirmed that Q and other polyphenols are potential P-gp expression and transport inhibitors which might sensitize resistant tumour cells to various drugs, including DB [27,34,36,37]. There are also available raports in which is described stimulation of the transport by some flavonoids, e.g., in cells of intestinal carcinoma resistant to DB [38,39]. Nevertheless, polyphenols do not act in the same way on all cells. Q exhibits pleiotropic properties depending on several conditions, e.g., type of tumour, redox status of cells, ATP concentration and/or applied time of Q and its concentration [40,41,42]. A 90% decrease in ATP-ase activity of P-gp was noted in MDR cells following application of 25 μM Q, while the same concentration was unable to affect ATP-ases of normal cells. Moreover, Q selectively influences kinases which participate in P-gp phosphorylation, e.g. protein kinase C, which manifests particularly high activity in MDR cells. Low Q concentrations might inhibit action of this type enzymes in tumour cells although no such effect has been noted yet in normal cells [21,42,43,44,45,46]. Q is also known to be capable of inhibiting the process of P-gp maturation in neoplastic cells such as molecule phosphorylation and its stabilization in the membrane [46]. Many data showed that Q not only exerts any inhibitory effect on transporters in normal cells and the important physiological barriers, but that it also protects healthy tissues from damage frequently accompanying action of a cytostatic drug [40,44,47,48,49]. The mutagenic effects on normal cells described by some authors might be linked to administration of very high doses. Low concentrations of Q affect expression of genes which products protect healthy cells from damaging factors, such as free radicals, heavy metals or thermal shock [50,51,52,53]. 

## Conclusions

Results of our studies confirmed that Q inhibits expression and function of P-gp in the RDB pancreatic carcinoma cell line. It is also effective in decreasing the expression of gene *ABCB1 *in both studied cell lines. Thus, Q may be considered as a potential sensitizer of neoplastic cells resistant to chemioterapeutics.

## Experimental

### Cell lines

The *in vitro* studies were performed on cell lines of human pancreatic carcinoma sensitive (P) and resistant (RDB) to daunorubicin, EPP85-181P and EPP85-181RDB respectively. The cell lines were obtained from Institute of Pathology, University Hospital Charite in Berlin where they had been established by *in vitro* exposure to daunorubicin (Farmitalia Carlo Erba, Freiburg, Germany). The cells were grown in Leibovitz's L-15 Medium (SIGMA, Germany) suplemented with 10% FBS, 1 mM L-glutamine, 80 IE/L insulin, 6.25 mg/L fetuin, 2.5 mg/L transferrin, 1.1 g/L NaHCO_3_, 1 g/L glucose, 1% minimal essential vitamins (SIGMA, Germany). The cell culture of both cell lines followed the description of Lage *et al*. [54].

### Calculation of resistance index for Q on the basis of cytotoxic tests

All proliferation tests were based on a colorimetric technique using sulphorhodamine B (SRB) dye, as described by Skehan *et al*. [55]. Substances used in the tests, quercetin (Q) and daunorubicin (DB), and the remaining reagents were purchased from SIGMA (Germany). The absorbance was read at the wavelength of 564 nm using a microplate-reader (ELX-800, BIO-TEK, USA). Concentrations of Q added 24h following starting the test: 1 µM, 2 µM, 3 µM, 6 µM, 12 µM, 25 µM and 50 µM. Q was dissolved in ethyl alcohol (concentration ≤ 0,12%), the adequate tests for the solvent were made before the further experiments.The absorbance in cultures with tested substances was read after 72 h after administration of the drugs. The cytotoxicity curves following 72 h (3 independent measurements) allowed to read out IC_50_ values and, then, Q resistance factor was calculated as follows: RF = IC_50_ for RDB/IC_50_ for P cells [36]. The cytotoxicity results permitted to select three concentrations of Q for further studies.

### Immunocytochemical analysis of P-gp expression

Cells of the two cell lines were transferred to diagnostic slides at 2 × 10^4^ cells/mL (three repetitions), after 24 h the tested substances were added: Q: 3 µM (Q3), 6 µM (Q6), 12 µM (Q12); DB: K1 (0.043 µM), K2 (0.43 µM), the concentration corresponding to the so called therapeutic dose, reflecting concentration of the cytostatic drug in patient’s blood two hours after its administration (for DB it amounted to 0.25 mg/mL), and K3 (4.3 µM); drug combinations: Q3/K1, Q3/K2, Q6/K1, Q6/K2, Q12/K1, Q12/K2; control (no drugs).

Following 72 h of incubation the cells were fixed in a cold (-20 °C) methanol-acetone (1:1) mixture for 15 min at the temperature of 4 °C and dried. Immunocytochemical detection of P-gp expression in cell membrane took advantage of LSAB+ System-HRP kit (Dako Cytomation, Denmark) and monoclonal P-gp-specific antibody, clone C-219 (Alexis Biochemicals, Germany). At the terminal stage of the immunocytochemical reaction DAB was used as a chromogen. Analysis of results was conducted under a light microscope (Olympus BX41, Japan). The percentage of cells was appraised which manifested P-gp expression among all cells in in five representative microscope fields (magnification 200×) for every well in a slide taking into account six wells and three independent repetitions.

### Western blotting analysis of P-gp

Changes in P-gp expression in RDB line were examined after treatment with the Q12 concentration, as compared to the control. Additionally the amount of P-gp was examined in P line. Cells were cultured in flasks of 75 cm^2 ^surface. Q12 was added for 72 h, then the cells were trypsinized, centrifuged from tripsin and medium, resuspended in PBS and scored in a Bürker haemocytometer.For each test 1.5–2 × 10^7^ exponentially grown cancer cell lines were washed in ice cold PBS and lysed on ice with RIPA buffer (50mM Tris-Cl pH 8.0, 150 mM NaCl, 0.1% SDS, 1% Igepal CA-630, Sigma, and 0.5% sodium deoxycholate) containing protease inhibitor cocktail (Sigma) and 0.5 mM PMSF. The cell debris were removed by centrifugation at 12 000 × g, 10 min. Protein concentration in resulting supernatant was measured using BCA method (Thermo-Pierce). Cell extracts were mixed with SDS sample buffer (250 mM TRIS pH 6.8, 40% glycerol, 20% (v/v) β-mercaptoethanol, 100 mM DTT, 0.33 mg/mL bromophenol blue, 8% SDS) and incubated on ice for 30 min to avoid membrane protein precipitation. Equal amounts of protein samples (20 μg per gel lane) were separated in 7% SDS-PAGE according to Laemmli [56], and blotted onto nitrocellulose membrane. Pgp expression was detected after incubation with Pgp-specific monoclonal antibody C219 (Alexis Biochemicals) using the chemiluminescence HRP detection substrate (Bio-Rad), and visualized with Chemi-Doc XRS Molecular Imager (Bio-Rad). To ensure equal protein loading blots were stripped and reprobed with mouse monoclonals to β-actin (ab8224, Abcam). Optical density measurements of the protein bands in immunoblots were performed with the QuantityOne software (Bio-Rad). Protein content in Pgp bands was normalized according to the actin content in each lane.

### Transport function of P-gp

Alterations in transport function of P-gp under effect of Q were examined using calcein AM (CAM) assay. RDB cells were transferred to 96-well plates, at 800 cells per well. After 24 h wells with resistant cell lines were supplemented with Q and DB for the subsequent 72 h, at the following combinations (every in six copies, three repetitions): Q3, Q6, Q12, K1, K2, K3, Q3/K1, Q3/K2, Q3/K3, Q6/K1, Q6/K2, Q6/K3, Q12/K1, Q12/K2, Q12/K3. Some wells was left with no additions, as a control. P line served for the control for calculation of retention coefficient. Retention of CAM was tested using Vybrant Multidrug Resistance Assay Kit (Molecular Probes, USA) according to the procedure given by the manufacturer [57]. The fluorescence was measured using a microspectrophotometer (Victor 2, USA) at 494/517 nm (Abs/Em). Values of fluorescence in P cell line (not treated with Q or DB) were used to calculate CAM retention coefficient for every applied drug combination in RDB line, according to the following formula: retention of CAM for RDB = [fluorescence of RDB cells treated with Q/fluorescence of P cells not treated with Q] × 100 [57].

### Examination of ABCB1 gene expression

Changes in *ABCB1 *gene expression in P and RDB cell lines were examined for the Q12 concentration, as compared to the control. Cells were cultured in flasks of 25 cm^2 ^surface, two flasks per each cell line and, then, concentration Q12 was added to one of the flask pair leaving the other one as the control. Following 72 h incubation the cells were trypsinized, suspended in PBS, spun down (3×), resuspended in a defined volume of PBS and scored in a Bürker haemocytometer. Then, the cells were again suspended in PBS. RNA was isolated using the RNa-queous-4PCR kit (Ambion, UK) as described by the manufacturer. Reverse transcription was performed using High Capacity cDNA RT kit (Applied Biosystems, USA) and a thermal cycler (Thermal Cycler MJ Research PTC-200, Bio-Rad, USA). Examination of changes in expression of *ABCB1* gene (Applied Biosystems, USA) was detected using real-time PCR. *GAPDH* (Applied Biosystems, USA) was applied as the normalizing gene, against which changes in the examined gene expression was compared. Real-time PCR was applied, using light-cycler’s 7900HT Fast Real-Time PCR System and SDS 2.3 software (Applied Biosystems, USA). Gene expression was analyzed using relative quantification RQ method and RQ Manager 1.2 software (Applied Biosystems, USA). RQ estimates difference at the level of gene expression against a calibrator (RQ of the calibrator = 1). P cell line not treated with Q and RDB cell line not treated with Q (as a comparison) were used as calibrators. The analysis was conducted employing the standard formula: RQ = 2^-∆∆Ct^, (where ∆∆Ct = ∆Ct for the sample - ∆Ct for the calibrator). The graphs were made in the logarithmic scale using RQ Manager 1.2 software.

### Statistical analysis

Statistical analysis of the results employed Student’s *t-*test (Mann-Whitney’s test) and SPSS 14.Pl software (SPSS Inc. USA). The results were regarded statistically significant at p < 0.01.
